# Upregulation of stomatin is associated with poor prognosis and promotes tumor progression of orbital diffuse large B-cell lymphoma

**DOI:** 10.3389/fonc.2025.1596614

**Published:** 2025-05-23

**Authors:** Wen Chen, Jingyi Chi, Weichen Song, Xiuyun Li, Jinlei Ma, Xinyu Liu, Lihua Xiao, Wenwen Zhu

**Affiliations:** ^1^ Department of Ophthalmology, Affiliated Hospital of Shandong Second Medical University, Weifang, Shandong, China; ^2^ Clinical Research Center, Affiliated Hospital of Shandong Second Medical University, Weifang, Shandong, China; ^3^ Department of Orbital Diseases, The Third Medical Center of Chinese PLA General Hospital, Beijing, China

**Keywords:** orbital diffuse large B-cell lymphoma, stomatin, cell proliferation, cell invasion, cell migration, cell apoptosis

## Abstract

**Introduction:**

Diffuse large B-cell lymphoma (DLBCL) is an aggressive subtype of non-Hodgkin’s lymphoma that predominantly affects the elderly and carries a poor prognosis. When arising in the orbit, DLBCL is characterized by rapid growth, high invasiveness, and a risk of severe vision loss. Despite the use of the R-CHOP regimen, long-term survival outcomes remain suboptimal, highlighting the need for new prognostic biomarkers and therapeutic targets.

**Methods:**

We explored stomatin as a potential prognostic biomarker and therapeutic target for orbital DLBCL. Stomatin expression was analyzed using the GEO database (GSE83632), and Mendelian randomization (MR) analysis was conducted to assess its causal relationship with DLBCL. Immunohistochemistry (IHC) was performed on orbital DLBCL specimens to evaluate stomatin expression. The functional role of stomatin was examined through siRNA-mediated knockdown in DLBCL cell lines, followed by validation using quantitative RT-PCR and Western blot. Cell proliferation, invasion, migration, and apoptosis were assessed using CCK-8, Transwell assays, and flow cytometry.

**Results:**

Database analysis revealed elevated stomatin expression in DLBCL, and MR analysis suggested a positive causal association with disease development. IHC confirmed significantly increased stomatin expression in orbital DLBCL tissues, which was associated with poor prognosis based on survival analysis. Vitro assays demonstrated that stomatin knockdown significantly inhibited cell proliferation, migration, and invasion while promoting apoptosis.

**Discussion:**

Our findings indicate that stomatin contributes to orbital DLBCL progression and is associated with adverse clinical outcomes. Stomatin may serve as both a prognostic biomarker and a potential therapeutic target for this malignancy.

## 1 Introduction

Orbital lymphoma (OL) is the most common primary malignant tumor in the orbit, originating from lymphoid tissues within or surrounding the orbital region (1, 2). It accounts for approximately 55%–67% of orbital tumors, with incidence rates rising globally (3). Diffuse large B-cell lymphoma (DLBCL), the most common of non-Hodgkin’s lymphoma worldwide (4, 5),is the most frequent aggressive histological subtype of OL, comprising approximately 13.4% of cases (6). DLBCL is divided into two subtypes based on cell origin: Germinal Center B-cells (GCB) and Non-GCBs. Non-GCB subtype is more common, accounting for 59% to 75% of cases (7).DLBCL primarily affects adults, with incidence increasing with age (8). It is highly malignant and aggressive (8), especially in cases involving the orbital region, presenting significant clinical challenges. However, there is currently no effective treatment. Patients who undergo surgical resection often suffer from recurrence and metastasis. About 60% of patients only achieve remission with the standard R-CHOP chemotherapy regimen (including rituximab, cyclophosphamide, doxorubicin, vincristine, and prednisone). About 30%–40% of patients respond poorly, experiencing early relapse, drug resistance, or even mortality (9). These challenges highlight the urgent need for novel therapeutic targets to improve survival outcomes for DLBCL patients.

Stomatin is an integral protein found in the plasma membrane of human red blood cells (10). It was first identified through studies on hereditary stomatocytosis, which is a type of hemolytic anemia (11). Stomatin is recognized as a crucial component of the cell membrane, playing key roles in regulating the activity of various channels and transporters (12–14), forming lipid rafts, mediating lipid droplet formation (10, 15, 16), and participating in the regulation of inflammatory responses (17–19). Recent studies have shown that stomatin is abnormally expressed in various malignant tumors, although the findings are inconsistent (20–24). However, the functions and mechanisms of stomatin in tumor tissues remain unclear.

Here, we investigated the potential association between orbital DLBCL and stomatin protein expression. First, bioinformatics analysis and Mendelian randomization (MR) studies were used to predict the potential significance of stomatin in orbital DLBCL. We also analyzed stomatin protein expression in orbital DLBCL tumor tissues and examined its relationship with pathological features. Then we explored the association between stomatin protein levels and the prognosis of patients with orbital DLBCL. Finally, we investigated the specific effects of stomatin on tumor growth and progression, aiming to reveal the underlying regulatory mechanisms.

## 2 Materials and methods

### 2.1 Analysis of differential gene expression

We collected the GSE83632 dataset from the GEO database, which includes expression data from 76 DLBCL samples and 83 normal samples. After standardization and log_2_ transformation, the data were analyzed for differences between normal and tumor tissues using the “limma” R package. Additionally, we calculated the expression levels of stomatin in various tissues.

### 2.2 MR analysis

The data for the stomatin gene were obtained from a comprehensive meta-analysis of blood eQTLs (25). Disease-related data were obtained from the OPEN GWAS database, specifically using the dataset labeled “Diffuse large B-cell lymphoma” (finn-b-C3_DLBCL). We established a significance threshold of 5 × 10–^8^ for the instrumental variables of the stomatin gene, including an LD coefficient (*r²*) of < 0.45 and independent SNPs with a physical distance greater than 10,000 kilobases (26). For our MR analysis, we used methods such as random effects inverse variance weighting (IVW), MR-Egger, and weighted median (WM) analyses. Primary results were derived using the IVW method, with statistical significance defined as *P* < 0.05. To assess pleiotropy, we employed MR-Egger regression, where a significant intercept *(P* < 0.05) indicates substantial pleiotropy (27). We also used Cochran’s Q statistic to test for heterogeneity; a significant Cochran’s Q (*P* < 0.05) indicates considerable heterogeneity in the effects of various genetic variations (28).

### 2.3 Patients

Between December 2008 and December 2014, thirty-six patients diagnosed with orbital DLBCL and treated with surgery at the Department of Orbital Diseases of the Third Medical Center of the Chinese PLA General Hospital (Beijing, China) and the Ophthalmology Center of Shandong Second Medical University Affiliated Hospital were enrolled in the study and followed up until January 2022. All studies involving human subjects were conducted following the principles of the Declaration of Helsinki and received approval from the Medical Ethics Committee of Weifang Medical University. All clinical samples were collected with written informed consent from the patients or their immediate family members.

Diagnosis of orbital DLBCL was confirmed by experienced pathologists for all patients. Among the patients, 29 (80.6%) had the ABC subtype and 7 (19.4%) had the GCB subtype. The cohort included 20 male and 16 female patients, aged 24 and 85 years (median age 60 years). Patients were followed up in person for at least 5 years to monitor recurrences.

### 2.4 Immunohistochemical staining

Surgically excised orbital tissues (classified as surgical discard) were fixed in 10% formalin for 12 hours and embedded in paraffin. Each sample was sectioned into two 4-μm slices and stained with an anti-stomatin antibody (Proteintech, Rosemont, USA). Erythrocyte staining served as an internal positive control. To verify the specificity of the staining, an immunohistochemical negative control was performed by replacing the primary antibody with PBS under the same experimental conditions. Two blinded senior pathologists independently assessed the stained sections. Stomatin-positive cells and staining intensity were evaluated in 10 randomly selected fields (100×magnification). The percentage of positive cells was categorized as 0 (≤5%), 1 (6%–25%), 2 (26%–50%), 3 (51%–75%), and 4 (75%). Staining intensity was scored as 1 (weak), 2 (moderate), and 3 (strong). The final score was calculated as the product of percentage and intensity scores, with low (final score <6) and high (final score ≥6) categories for stomatin expression (29).

### 2.5 Cell culture and transfection

OCI-Ly3 was cultured in OCI-Ly3 cell-specific medium (Pricella Life Science & Technology Co., Ltd, Wuhan, China), which contained 20% fetal bovine serum and 1% penicillin-streptomycin, mixed with RPMI 1640 medium. Cells were maintained at 37°C in a 5% CO_2_ atmosphere with saturated humidity. Small interfering RNA (siRNA) targeting stomatin (GenePharma, Shanghai, China) was transfected into OCI-Ly3 cells using Lipofectamine 3000 (Thermo Fisher Scientific, MA, USA). Negative control (NC) siRNA (GenePharma, Shanghai, China) was transfected into cells as the control group. Si-stom (sense 5′-GCAGUCUACUCUGGAUGAUTT-3′, antisense 5′-AUCAUCCAGAGUAGACUGCTT-3′), and NC siRNA (sense 5′-UUCUCCGAACGUGUCACGUTT-3′, antisense 5′-ACGUGACACGUUCGGAGAATT-3′) were designed and synthesized by GenePharma.

### 2.6 Quantitative reverse-transcription PCR

After 24 hours of transfection with siRNA negative control (NC) and siRNA targeting stomatin (si-stom), total RNA was extracted using TRnaZol Reagent (New Cell & Molecular Biotechnology, Suzhou, China). Reverse transcription was performed to synthesize cDNA using the Primescript Master Mix (Perfect Real Time) kit (Takara Bio Inc, Shiga, Japan). Quantitative Reverse-Transcription PCR (qRT-PCR) amplification was conducted using the TB Green Premix Ex Taq II kit (Takara Bio Inc, Shiga, Japan). Equal amounts of reverse transcription products were mixed with other components to form a 10μl reaction system. Stomatin mRNA expression in OCI-Ly3 cells was normalized to GAPDH as the internal reference gene. Specific primers were synthesized by GenePharma (Shanghai, China): stomatin, forward 5′-AAAGGTGGAGCGTGTGGAAA-3′, reverse 5′-CTTCGGCTGCAATAACCTTGG-3′; GAPDH, forward 5′-AAGCTCATTTCCTGGTATGACAA-3′, reverse 5′-CTTACTCCTTGGAGGCCATGT-3′.

### 2.7 Cell proliferation assay

After 48 hours of transfection with siRNA, NC cells and si-stom cells were seeded in 96-well plates at a density of 5 × 10^3^ cells per well, with a volume of 100 μl per well. Ten microliters of CCK-8 (Beyotime Biotechnology, Shanghai, China) was added to each well, and the plates were incubated at 37°C for 4 hours. The absorbance at 450 nm was measured using a microplate reader.

### 2.8 Transwell assay for cell migration and invasion

After transfection, NC and si-stom cells were collected by centrifugation, resuspended in RPMI-1640 serum-free medium (Gibco, Thermo Fisher Scientific, MA, USA), and adjusted to a density of 1.5 × 10^6^ cells/ml. A 200 μl aliquot of the cell suspension was then added to the Transwell chambers (Corning, New York, USA). The cells were incubated at 37°C for 48 hours, after which they were collected for migration analysis. For the invasion assay, cells were resuspended in RPMI-1640 serum-free medium and seeded onto a polycarbonate membrane pre-coated with a uniform layer of Matrigel (Corning, New York, USA) in the Transwell chambers. The cell density was adjusted to 1.5 × 10^6^ cells/ml, and 200 μl of the cell suspension was added to each chamber. The cells were incubated again at 37°C for 48 hours. After incubation, the cells were fixed in 4% paraformaldehyde (Solarbio, Beijing, China) for 10 minutes and stained with 0.1% crystal violet (Solarbio, Beijing, China) for 15 minutes. The stained cells were observed under an inverted microscope at 200× magnification and photographed for analysis.

### 2.9 Apoptosis assays

After OCI-Ly3 cells were transfected with siRNA, NC and si-stom cells were collected, refreshed with new medium, and re-cultured for 48 hours. Apoptosis in the NC and si-stom cells was then assessed using the Annexin V-FITC/7-AAD Fluorescence Double Staining Apoptosis Detection Kit (Pricella, Wuhan, China).

### 2.10 Western blot

After transfection of OCI-Ly3 cells, NC and si-stom cells were collected, and proteins were extracted using RIPA lysis buffer (Beyotime Biotechnology, Shanghai, China). The concentration of the target protein was determined using the BCA method, and equal amounts of protein were separated by SDS-PAGE. The proteins were transferred to a PVDF membrane at a constant current of 400 mA and blocked with blocking buffer (Boster, Wuhan, China). Membranes were incubated overnight with antibodies against stomatin (1:1000, Proteintech, Rosemont, USA) and β-actin (1:1000, Proteintech, Rosemont, USA). After washing with Tris-HCl-Tween buffered saline solution (TBST), the membranes were incubated at room temperature for 1 hour with horseradish peroxidase-conjugated goat anti-rabbit secondary antibody (Proteintech, Rosemont, USA). Finally, the membranes were developed using Super ECL Plus chemiluminescent substrate (Applygen, Beijing, China), and images were analyzed using Image J.

### 2.11 Statistical analysis

Data analysis was conducted using GraphPad Prism v10.0, SPSS 25, and ImageJ software. Statistical analyses included unpaired t-tests and two-way ANOVA, with significance levels indicated in the corresponding figures. Spearman correlation coefficients were calculated to evaluate the relationships between two variables. The strength of correlation was calculated (*r*). *P* value < 0.05 was considered statistically significant.

## 3 Results

### 3.1 Analysis of differential gene expression

**Figure 1:**
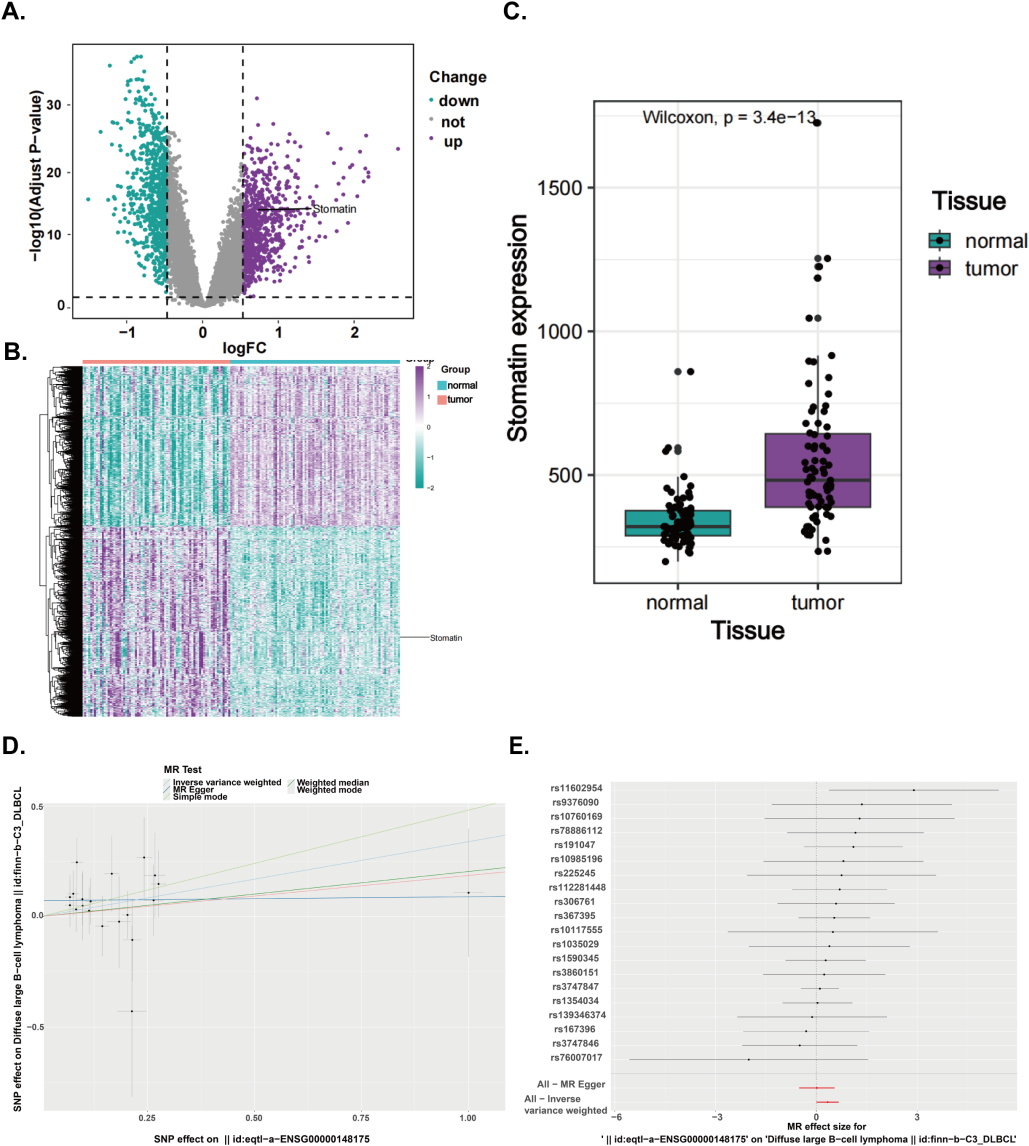
The expression difference analysis and MR analysis of stomatin were carried out, based on GSE83632 dataset and OPEN GWAS database. **(A)** Volcano plot of differentially expressed genes between normal and tumor tissue in GSE83632 (Adjust-p value <0.05 and |log_2_FC|≥0.5). **(B)** Heatmap of differentially expressed genes between normal and tumor tissue in GSE83632. **(C)** The expression levels of stomatin in DLBCL and normal tissues were analyzed by the GSE83632 dataset. **(D)** Sensitivity analysis for Mendelian randomization (scatter plot). **(E)** Sensitivity analysis for Mendelian randomization (forest plot).

To better understand the role of stomatin in different tumor types, we first conducted a pan-cancer analysis of the stomatin gene. The results showed that the expression levels of stomatin vary across different tumor types (**Supplementary Figure S1**). The goal of this study is to explore the specific expression pattern of stomatin in orbital DLBCL, particularly in comparison to other tumor types, where stomatin exhibits relatively higher expression levels. Differential expression analysis of the GSE83632 dataset identified 1531 differentially expressed genes (adjusted p-value < 0.05 and |log_2_FC| ≥ 0.5), and their expression is shown in a heatmap (**Figures 1A, B**). Among them, stomatin was highly expressed in tumor tissues, demonstrating a degree of specificity in DLBCL (**Figure 1C**).

### 3.2 MR analysis

To explore the causal relationship between the stomatin gene and DLBCL, we performed MR analysis. The aim of this analysis was to assess the potential causal relationship between stomatin and orbital DLBCL through genetic variation, thereby avoiding confounding factors that may exist in traditional observational studies. At the genome-wide level, we identified and screened 20 independent stomatin SNPs that were not in linkage disequilibrium. The IVW analysis showed a positive causal association between stomatin gene and DLBCL [OR = 1.40, 95% CI (1.01, 1.95), *P* = 0.04, which is statistically significant at *P* < 0.05]. As shown in **Supplementary Table S1**, the Q-pvalue from the IVW analysis was 0.91, indicating no evidence of heterogeneity. Additionally, the MR-Egger intercept test suggested that directional pleiotropy was unlikely to introduce bias (*P* = 0.14), confirming that pleiotropy was not present in our data (**Figures 1D, E**).

### 3.3 Patient outcomes and stomatin expression level correlated with clinical and pathological characteristics of orbital DLBCL

**Figure 2:**
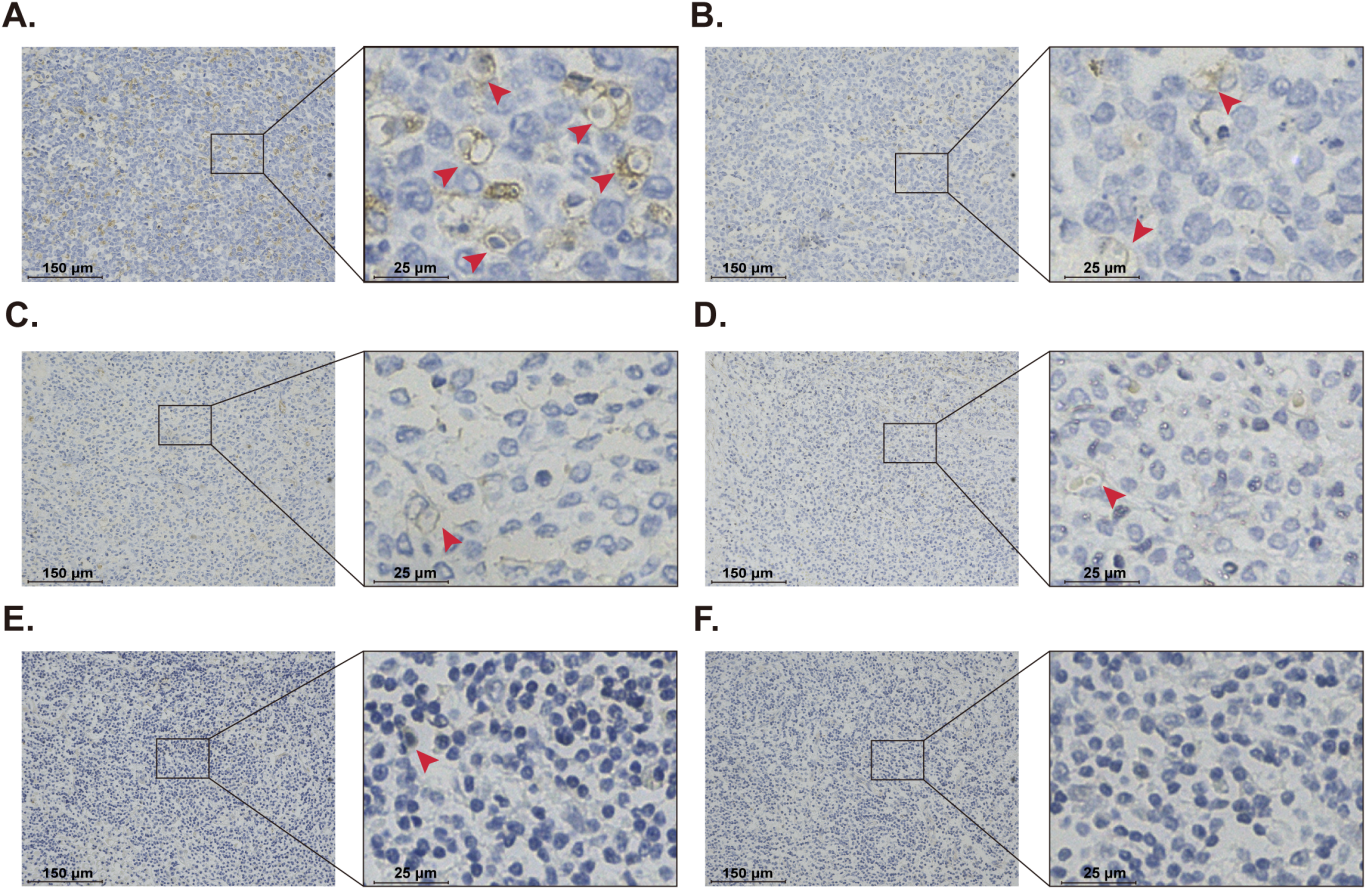
Immunohistochemical staining of stomatin in orbital diseases. **(A, B)** Orbital diffuse large B-cell lymphoma. **(C)** Orbital MALT lymphoma. **(D)** Orbital follicular lymphoma. **(E)** Orbital lymphoreactive hyperplasia. **(F)** Inflammatory pseudotumor.

After a minimum follow-up of 5 years, 50% of patients (18/36) experienced tumor recurrence, and 44.4% (16/36) died from their tumors. The median disease-free survival was 27.5 months (range, 8–70 months), and the median overall survival was 49 months (range, 9–89 months). Only three patients received Rituximab treatment, of whom two patients received the R-CHOP regimen and one patient received the R-CVP regimen. None of the patients has a poor response to Rituximab. Immunohistochemical analysis showed that stomatin protein expression was localized to the cell membrane in all orbital DLBCL tissues, with varying rates of positive expression (**Figures 2A, B**). The percentage of the stomatin stained cells varied from 15% to 56% in these samples. The median proportion of stomatin stained cells was 37.5%. Out of 36 patients with ocular DLBCL, 18 showed low expression (final score <6) and 18 showed high expression (final score ≥6) of stomatin. Intraorbital tumor sections of patients with orbital MALT lymphoma, follicular lymphoma, orbital lymphoreactive hyperplasia, and orbital inflammatory pseudotumor were also stained. In orbital MALT lymphoma and follicular lymphoma, staining intensity was weaker and the proportion of stomatin stained cells was less than 10% (**Figures 2C, D**). In orbital lymphoreactive hyperplasia and orbital inflammatory pseudotumor, the proportion of stomatin-positive cells was less than 5% (**Figures 2E, F**).

As shown in **Table 1**, there were 18 patients each with high and low stomatin expression. Stomatin expression was significantly higher in Ann Arbor stage II-IV tumors compared to stage I tumors (*P*=0.002). Furthermore, patients with low stomatin expression had a significantly lower recurrence rate than those with high stomatin expression *(P*=0.002). Additionally, high stomatin expression was associated with higher lactate dehydrogenase (LDH) levels (*P*=0.000).

**Table 1:** Stomatin expression and its relationship with clinical pathological characteristics of 36 orbital DLBCL patients.

Characteristic	n	Low expression (n)	Low expression (%)	High expression (n)	High expression (%)	*P*-value
Age (years)						0.765
≤60	18	10	55.6	8	44.4	
>60	18	8	44.4	10	55.6	
Sex						0.738
Male	20	8	40.0	12	60.0	
Female	16	10	62.5	6	37.5	
Side of involvement						0.052
Monocular	30	16	53.3	14	46.7	
Binocular	6	2	33.3	4	66.7	
Tumor size (mm³)						0.086
<12	20	13	65.0	7	35.0	
≥12	16	5	31.3	11	68.8	
LDH						0.000
≤240 U/L	30	18	60.0	12	40.0	
>240 U/L	6	0	0.0	6	100.0	
ESR						0.628
Normal	26	14	53.8	12	46.2	
Above normal	10	4	40.0	6	60.0	
Serum albumin (g/L)						0.915
≤35	11	6	54.5	5	45.5	
>35	25	12	48.0	13	52.0	
B symptoms						0.189
Absent	31	17	54.8	14	45.2	
Present	5	1	20.0	4	80.0	
Ann Arbor stage						0.002
I	18	14	77.8	4	22.2	
II/III/IV	18	4	22.2	14	77.8	
Recurrence						0.002
Absent	18	16	88.9	2	11.1	
Present	18	2	11.1	16	88.9	

LDH, lactate dehydrogenase; ESR, erythrocyte-sedimentation rate.

**Figure 3:**
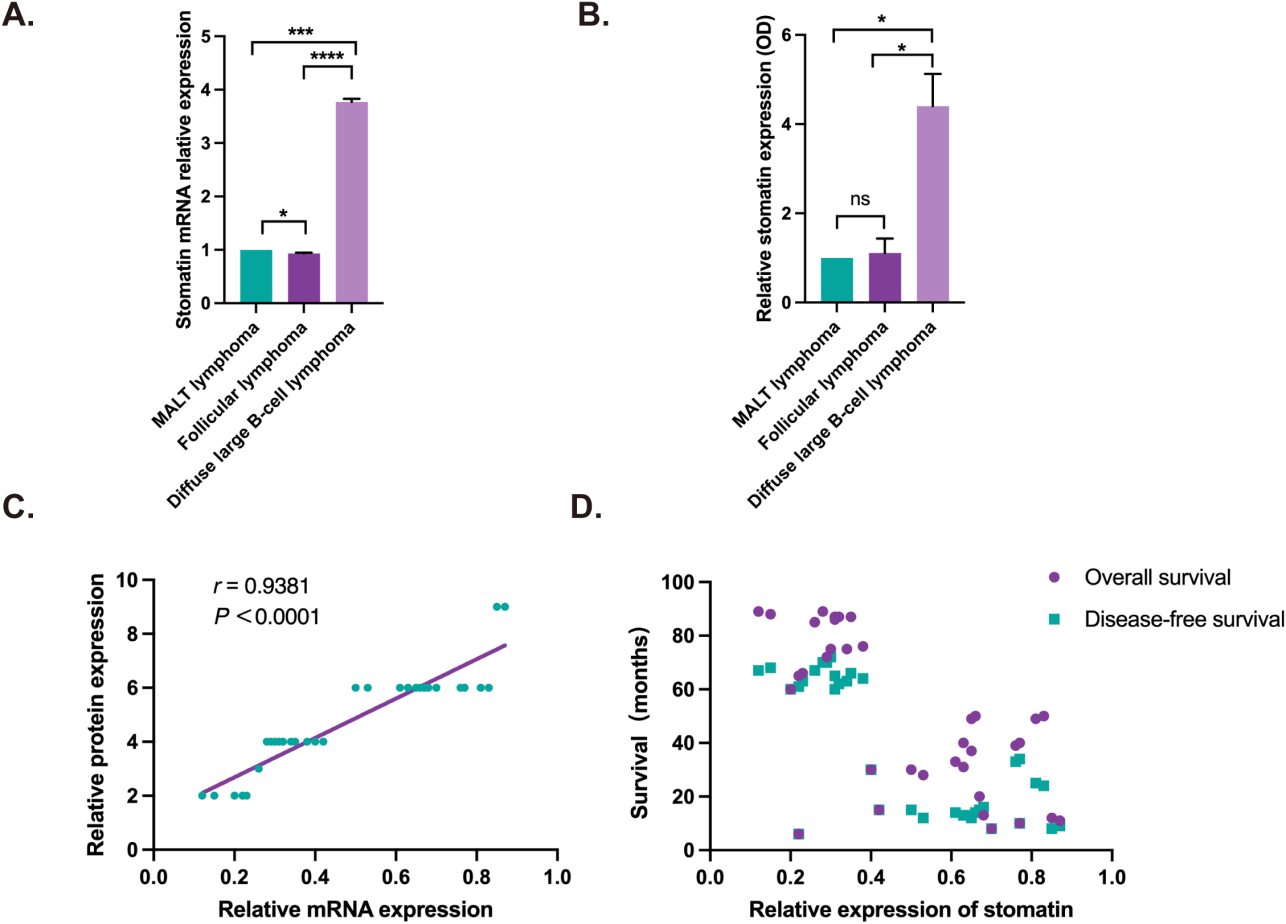
Stomatin mRNA and protein expression in orbital lymphoma and their correlation analysis. **(A)** Stomatin mRNA expression in orbital lymphoma tissue. **(B)** Stomatin protein expression in orbital lymphoma, quantified based on IHC results. **(C)** Correlation between stomatin mRNA expression and protein expression in orbital DLBCL tissue, with protein expression based on IHC scoring. **(D)** Correlation analysis showing the association between stomatin mRNA expression and overall survival (OS) as well as disease-free survival (DFS) in patients with orbital DLBCL. Correlation analysis revealed a significant negative correlation between stomatin mRNA expression and OS (*r* = -0.6272, *P* < 0.0001) as well as DFS *(r* = -0.6479, *P* < 0.0001). *P < 0.05, *** P < 0.001, **** P < 0.0001. NS, Not Significant.

We also examined the expression of stomatin mRNA using qRT-PCR in the biopsied specimen of orbital lymphoma. The expression of stomatin mRNA and protein in orbital tumor tissue of patients with orbital DLBCL was significantly higher than that of orbital MALT lymphoma and orbital follicular lymphoma (*P*<0.05) (**Figures 3A, B**). It was found that the expression of stomatin mRNA is closely related to the expression of stomatin protein (*r*=0.9381, *P*<0.0001) (**Figure 3C**). The expression of stomatin mRNA was closely related to overall survival (*r* = -0.6272, *P*<0.0001) and disease-free survival (*r* = -0.6479, *P*<0.0001) (**Figure 3D**).

### 3.4 Survival analysis

**Figure 4:**
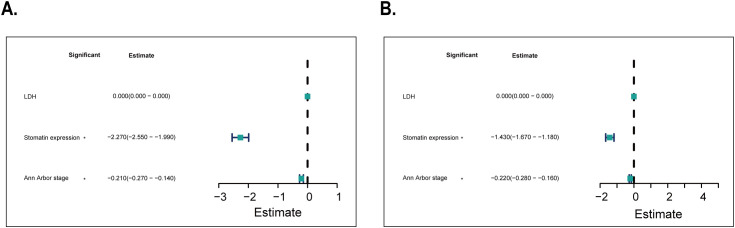
Results of multivariate survival analysis adjusted by Bayesian regression model. **(A)** Multivariate analysis results of disease-free survival in 36 patients with orbital DLBCL. **(B)** Multivariate analysis results of overall survival in 36 patients with orbital DLBCL. (LDH, lactate dehydrogenase) *Р < 0.05.

**Figure 5:**
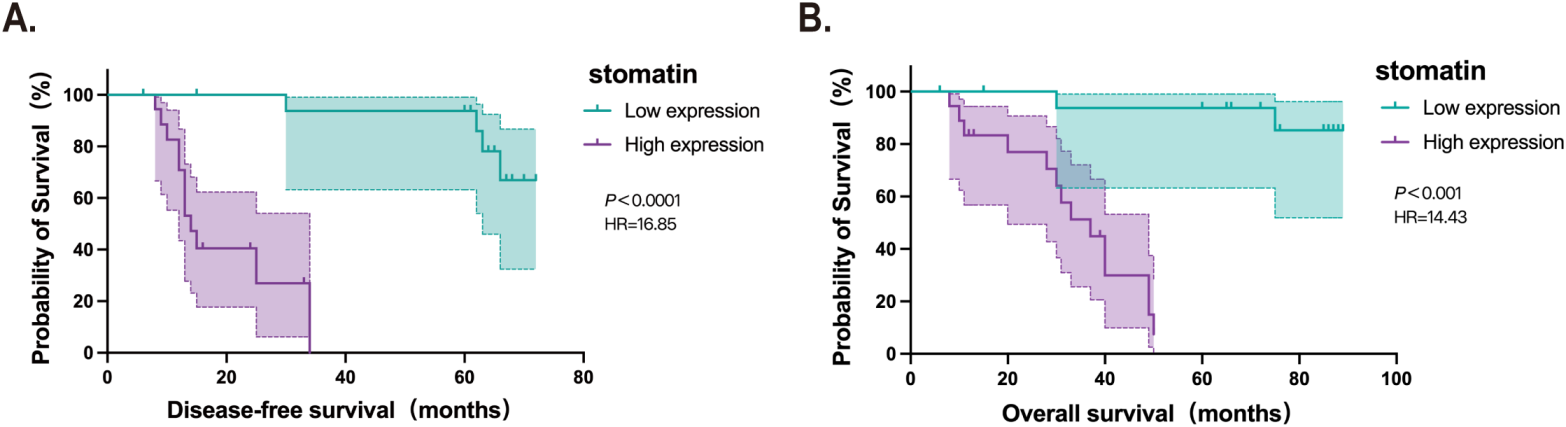
Survival analysis. **(A)** Disease-free survival of patients with low and high stomatin expression in orbital DLBCL. **(B)** Overall survival of patients with low and high stomatin expression in orbital DLBCL.

Univariate analysis showed significant associations between stomatin protein expression, Ann Arbor stage, and LDH levels with disease-free and overall survival in patients (**Table 2**). Multivariate analysis was first performed using a Cox regression model (**Table 3**) and further adjusted using a Bayesian regression model (**Supplementary Tables S2, S3**). The adjusted results indicated that Ann Arbor stage and stomatin expression were independent prognostic factors for both disease-free and overall survival (**Figure 4**). Survival analysis further revealed that patients with high stomatin expression had significantly shorter survival times than those with low expression (**Figure 5**).

**Table 2:** Univariate analysis of clinicopathological factors for disease-free and overall survival of 36 orbital DLBCL patients.

Factor	n	Recurrences	Disease-free survival rate	*P*-value	Deaths	Overall survival rate	*P*-value
Stomatin expression				0.000			0.000
Low level	18	4	77.8%		2	88.9%	
High level	18	12	33.3%		14	22.2%	
Age, years				0.726			0.157
≤60	18	9	50.0%		7	61.1%	
>60	18	7	61.1%		9	50.0%	
Sex				0.669			0.161
Male	20	9	55.0%		9	55.0%	
Female	16	7	56.3%		7	56.3%	
Side of involvement				0.246			0.386
Monocular	28	13	53.6%		12	57.1%	
Binocular	8	3	62.5%		4	50.0%	
Tumor size (mm^3^)				0.599			0.346
<12	20	9	55.0%		8	60.0%	
≥12	16	7	56.3%		8	50.0%	
LDH				0.000			0.000
≤240 U/L	30	10	66.7%		10	66.7%	
>240 U/L	6	6	0.0%		6	0.0%	
ESR				0.189			0.058
Normal	26	10	61.5%		10	61.5%	
Above normal	10	6	40.0%		6	40.0%	
Serum albumin (g/L)				0.743			0.907
≤35	10	5	50.0%		4	60.0%	
>35	26	11	57.7%		12	53.8%	
B symptoms				0.740			0.921
Absent	32	12	62.5%		12	62.5%	
Present	4	4	0.0%		4	0.0%	
Ann Arbor stage				0.000			0.001
I	18	5	72.2%		5	72.2%	
II/III/IV	18	11	38.9%		11	38.9%	

LDH, lactate dehydrogenase; ESR, erythrocyte-sedimentation rate.

**Table 3:** Multivariate analysis of clinicopathological factors for disease-free and overall survival of 36 orbital DLBCL patients.

Outcome	Variable	Category	Relative Risk	*P*-value
**Disease-free survival**	Stomatin expression	High level	6.143	0.040
		Low level		
	Ann Arbor stage	I	1.829	0.418
		II/III/IV		
	LDH	≤240 U/L	1.690	0.440
		>240 U/L		
**Overall survival**	Stomatin expression	High level	20.186	0.008
		Low level		
	Ann Arbor stage	I	1.631	0.437
		II/III/IV		
	LDH	≤240 U/L	1.289	0.649
		>240 U/L		

LDH, lactate dehydrogenase.

### 3.5 Cell transfection

**Figure 6:**
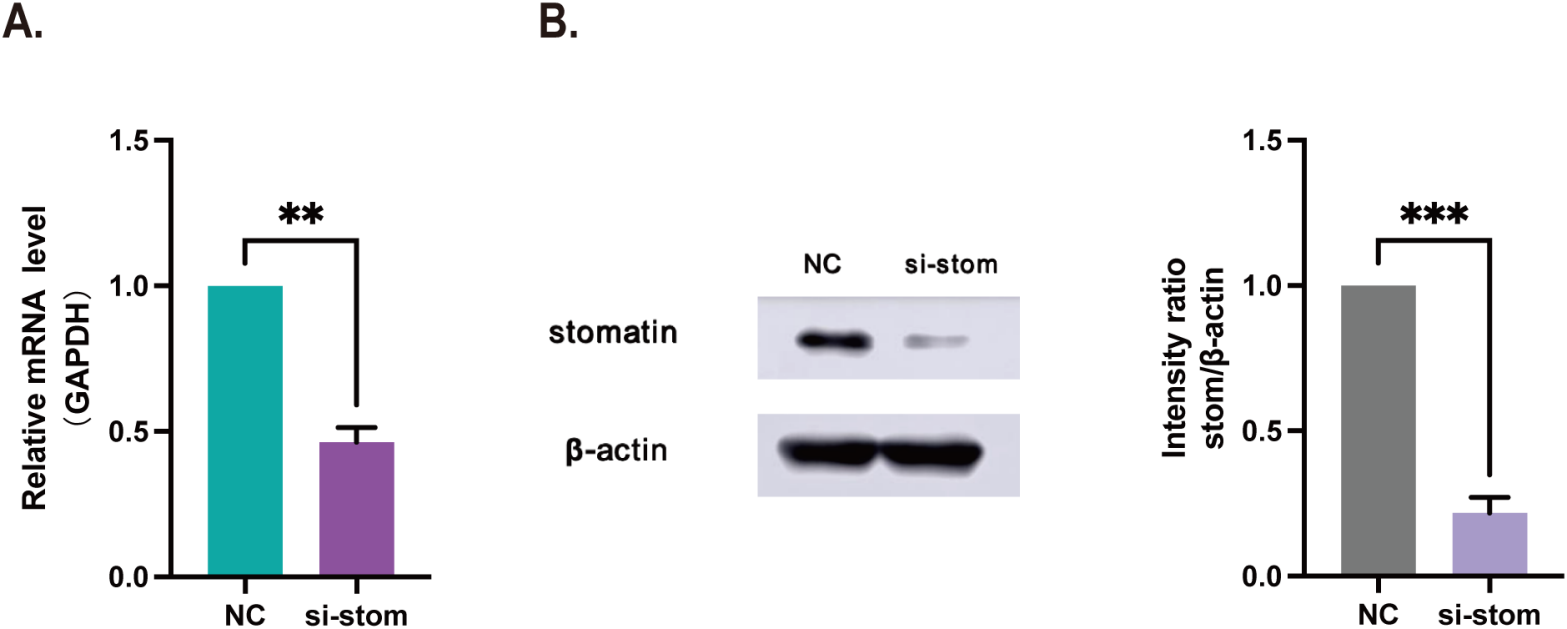
SiRNA transfection results. **(A)** OCI-Ly3 cells after transfection with si-stom by qRT-PCR. **(B)** OCI-Ly3 cells after transfection with si-stom by Western blot. NC, negative control; si-stom, siRNA targeting stomatin. **P < 0.01, ***P < 0.001.

To verify the promoting effect of stomatin protein on orbital DLBCL, we performed *in vitro* experiments to knock down the expression of stomatin in two DLBCL cell line. The transfection efficiency was validated at both the molecular and protein levels. The results of qRT-PCR and Western blot analyses showed that the expression of stomatin at both the mRNA and protein levels was significantly reduced in si-stom cells compared to NC cells after siRNA transfection. The difference was statistically significant (**Figure 6**).

### 3.6 Cell proliferation ability

**Figure 7:**
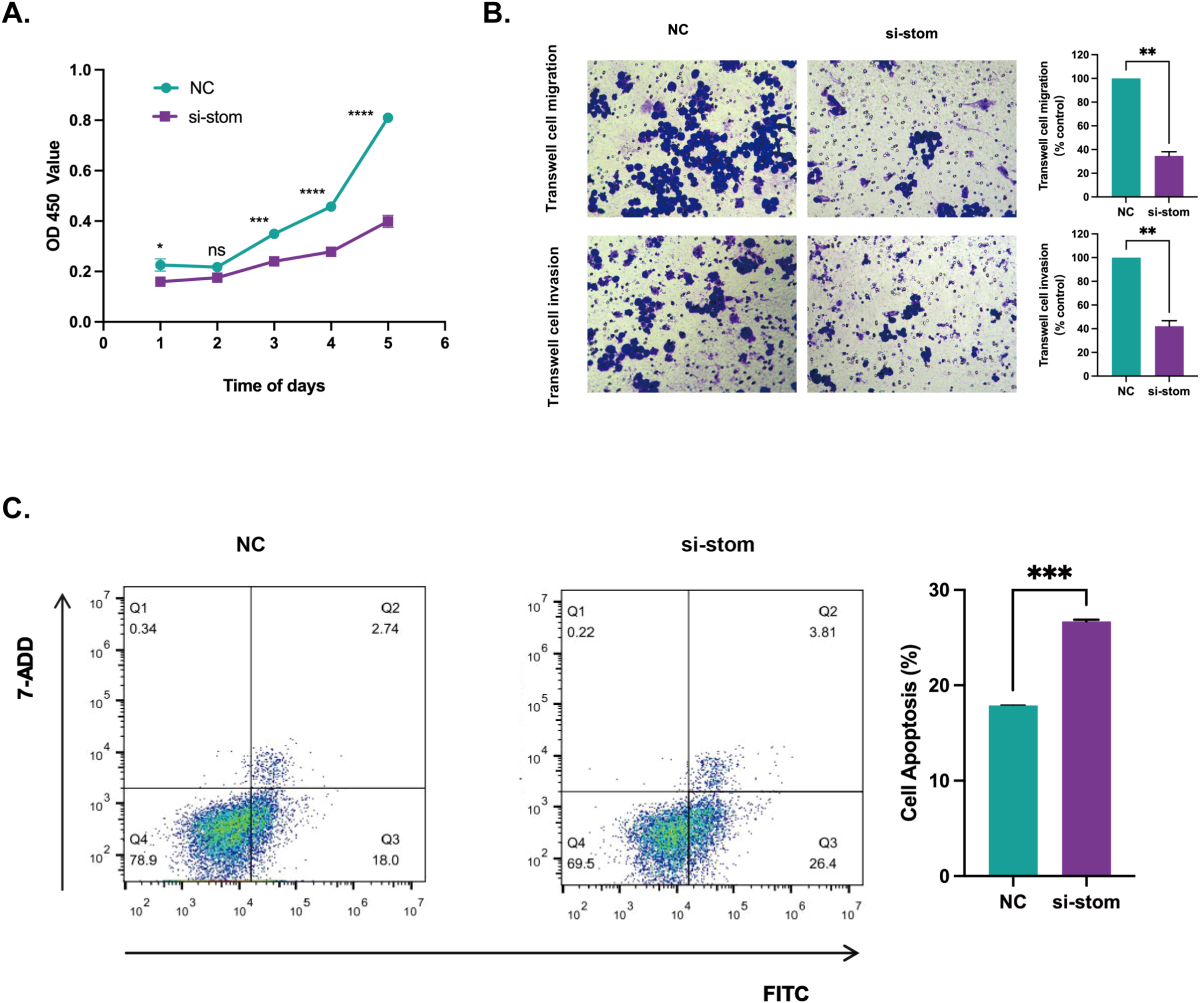
The effect of stomatin gene knockdown on cell phenotype. **(A)** The effect of stomatin gene knockdown on OCI-Ly3 cell proliferation assessed by the CCK-8 assay. **(B)** The effect of stomatin knockdown on OCI-Ly3 cell migration and invasion assessed by transwell assay. **(C)** The effect of stomatin knockdown on OCI-Ly3 cell apoptosis assessed by flow cytometry. *P < 0.05, **P < 0.01, ***P < 0.001, ****P < 0.0001. NS, Not Significant.

We used the CCK-8 assay to assess the cell proliferation ability of si-stom and NC cells after transfection. The results are shown in **Figure 7A**. The proliferation ability of si-stom cells was significantly reduced compared to NC cells within 5 days following stomatin gene knock down, indicating that stomatin has a promoting effect on the growth of DLBCL cells.

### 3.7 Cell invasion and migration ability

To further verify the promoting effect of stomatin protein on DLBCL, we conducted transwell experiments to assess the invasion and migration abilities of cells. The results, as shown in **Figure 7B**, indicate that after transfection, the invasion and migration abilities of si-stom cells were significantly weakened. These findings suggest that stomatin promotes the invasion and migration capabilities of DLBCL.

### 3.8 Cell apoptosis

To further elucidate the promoting effect of stomatin protein on DLBCL, we stained cells transfected with the stomatin gene using Annexin V-FITC and 7-AAD, and detected the effect of stomatin protein on cell apoptosis using flow cytometry. As shown in **Figure 7C**, Q1 represents necrotic cells, Q2 represents late apoptotic cells, Q3 represents early apoptotic cells, and Q4 represents live cells. The results showed that the apoptosis rate of si-stom cells was 30.21%, while the control group had an apoptosis rate of 20.74%. There was a significant difference between the experimental group and the control group, and the difference was statistically significant (*P* < 0.05).

## 4 Discussion

This study provides comprehensive evidence that stomatin promotes the growth and development of orbital DLBCL. Bioinformatics analysis revealed that stomatin is overexpressed in DLBCL patients relative to healthy individuals, highlighting its role in tumor promotion. MR analysis establishes a positive causal relationship between stomatin and DLBCL, further corroborating the role of stomatin in tumor growth and progression. Building on these findings, we conducted a series of *in vitro* experiments. The results regarding cell proliferation, invasion, migration, and apoptosis indicated that stomatin enhances tumor cell progression. We also found that stomatin expression is lower in orbital tumor tissues of patients with other types of B-cell lymphoma, suggesting its high specificity in orbital DLBCL. Our findings emphasize the importance of stomatin in DLBCL and suggest its potential as a therapeutic target.

Current research has identified abnormal stomatin expression in several types of malignant tumors; however, findings on its role remain inconsistent. In breast cancer, several studies suggest that reduced stomatin expression may be linked to poorer prognosis (21). Conversely, our findings indicate that high stomatin expression levels in orbital DLBCL tissues may be predictive of poor prognosis and are significantly associated with higher recurrence rates. These results suggest that stomatin could serve as a potential prognostic marker for patients with orbital DLBCL. In summary, these findings underscore the potential utility of stomatin as a prognostic marker across various cancer types.

Stomatin is a member of the stomain protein family along with stomatin-like protein 1 (SLP-1), stomatin-like protein 2 (SLP-2), stomatin-like protein 3 (SLP-3) and podocin, and stomatin also belongs to another superfamily, SPFH (Stomatin, Prohibitin, Flotillin, HflK/HflC domain), which share sequence similarities (10, 30, 31). Findings suggest that each member of the SPFH superfamily exerts a different role in tumor cells in a cell type-dependent manner (24). For example, SLP-2 can promote the progression of various tumors, such as pancreatic cancer, ovarian cancer, and gastric cancer, and its high expression is closely associated with poor prognosis (32–35). Interestingly, and this is coincident with the results of the current study, stomatin promotes the progression of DLBCL, and its overexpression in tumors serves as a marker of poor prognosis. Reports have claimed that SPFH superfamily member prohibitin, on the other hand, has both promotional and inhibitory effects on tumors (36–38). As for flotillin, another member of the SPFH superfamily, it has been reported that it is overexpressed in many cancers and promotes tumor growth (39–41). However, there is also a table in the literature showing that flotillin may have an inhibitory effect on cancers (42). In summary, the reasons for the discrepancy between the existing reports that stomatin can inhibit cancer development and the present study (20–24) may be the same as those of other members of the SPFH superfamily, which have different effects in different cellular environments, and it is worthwhile for us to continue to explore the specific mechanisms of the reasons for the discrepancy.

Metabolic reprogramming is an important way for tumors to maintain malignant vitality (43), and reprogramming lipid metabolism is critical for tumor growth and development (44–46). Studies have shown that reprogramming lipid metabolism plays an important role in the development of DLBCL, suggesting that targeting DLBCL lipid metabolism may be an important direction for the development of novel anticancer therapeutic strategies (47). Current research indicates that stomatin is involved in various physiological activities, including the generation and maturation of adipocytes (15). Given the potential role of stomatin in lipid metabolism, we hypothesize that stomatin may play a crucial role in reprogramming lipid metabolism in DLBCL. Therefore, targeting stomatin or its mediated lipid metabolism pathways may represent an important direction for the development of novel anticancer drugs and therapeutic regimens. Future studies could further explore the specific mechanisms of action of stomatin through lipid metabolic pathways in DLBCL, as well as evaluate the efficacy and safety of stomatin-based anticancer therapeutic strategies.

Given the potential role of stomatin in lipid metabolism, we hypothesize that stomatin may play a crucial role in reprogramming lipid metabolism in DLBCL. Therefore, targeting stomatin or its mediated lipid metabolism pathways may represent an important direction for the development of novel anticancer drugs and therapeutic regimens. Future studies could further explore the specific mechanisms of action of stomatin through lipid metabolic pathways in DLBCL, as well as evaluate the efficacy and safety of stomatin-based anticancer therapeutic strategies.

There are some limitations in our study. First, we did not validate the *in vivo* experiments in our present study. Because stomatin knockout mice do not develop an obvious phenotype in the physiological state (20, 48) and the mouse orbital tumorigenic model is not well developed. Currently, there is limited research on the establishment of intraocular lymphoma models (49–51), and no reports have been found on the establishment of an animal model for orbital lymphomas. The subcutaneous tumor-bearing mouse model is widely used in cancer research, as it allows for the validation of tumor biological characteristics in an *in vivo* environment. However, the space for tumor growth is very different between the subcutaneous area and the orbit. This limits the application of subcutaneous tumor-bearing mouse model in orbital tumor research. Second, we have only examined the expression of stomatin in orbital DLBCL tumor tissues. Due to the limited samples, we have not yet observed stomatin expression in DLBCL at other body sites. This will be investigated in future studies.

In conclusion, our study reveals that stomatin is overexpression in orbital DLBCL. Stomatin could promote tumor progression and maybe stomatin is a good therapeutic target for orbital DLBCL.

## Data Availability

The datasets presented in this study can be found in online repositories. The names of the repository/repositories and accession number(s) can be found in the article/**Supplementary Material**.
