# Supplementary material for: Upregulation of stomatin is associated with poor prognosis and promotes tumor progression of orbital diffuse large B-cell lymphoma
Source: Front Oncol. 2025 May 23;15:1596614. doi: 10.3389/fonc.2025.1596614 (PMC12141002; doi:10.3389/fonc.2025.1596614)
Supplement: Supplementary file 1 [file DataSheet1.docx]

Supplementary Material

# Supplementary Figures and Tables

## Supplementary Figures


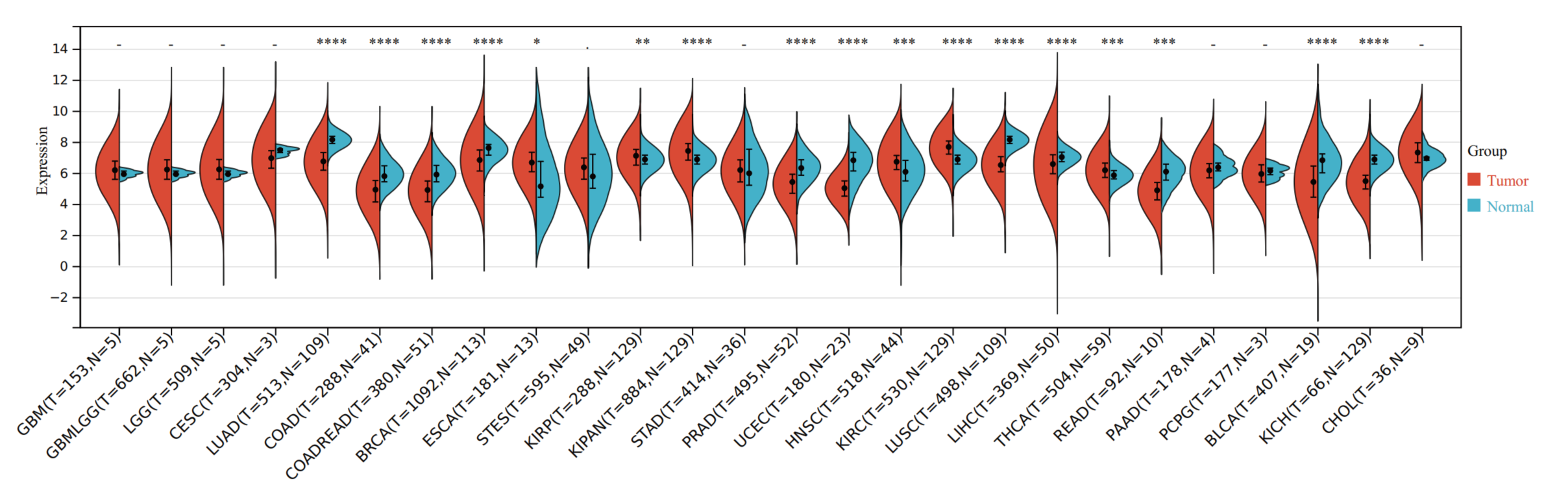


**Supplementary Figure 1.** Monogenic pan-cancer analysis. We downloaded the unified and standardized pan-cancer dataset: TCGA Pan-Cancer (PANCAN, N=10535, G=60499) from the UCSC (https://xenabrowser.net) database, and further extracted the expression data of ENSG00000148175 (stomatin) gene in each sample. We finally obtained the expression data of 26 cancer types. BLCA(Bladder Urothelial Carcinoma), BRCA (Breast invasive carcinoma), CESC(Cervical squamous cell carcinoma and endocervical adenocarcinoma), CHOL(Cholangiocarcinoma), COAD(Colon adenocarcinoma), COADREAD(Colon adenocarcinoma/Rectum adenocarcinoma Esophageal carcinoma), ESCA(Esophageal carcinoma) GBM(Glioblastoma multiforme), GBMLGG(Glioma), HNSC(Head and Neck squamous cell carcinoma), KICH(Kidney Chromo-phobe), KIPAN(Pan-kidney cohort KICH+KIRC+KIRP), KIRC(Kidney renal clear cell carcinoma), KIRP(Kidney renal papillary cell carcin-oma), LGG(Brain Lower Grade Glioma), LIHC(Liver hepatocellular carcinoma), LUAD(Lung adenocarcinoma), LUSC(Lung squamous cell carcinoma), PAAD(Pancreatic adenocarcinoma), PCPG(Pheochromocytoma and Paraganglioma), PRAD(Prostate adenocarcinoma), READ (Rectum adenocarcinoma), STAD(Stomach adenocarcinoma), STES(Stomach and Esophageal carcinoma), THCA(Thyroid carcinoma), UCEC(Uterine Corpus Endometrial Carcinoma). **P* < 0.05. ***P* < 0.01. ****P* < 0.001. *****P*< 0.0001.

## Supplementary Tables

| **Table S1.** Results of Mendelian randomization analysis. | | | | | |
| --- | --- | --- | --- | --- | --- |
| **exposure** | **IVW(OR, 95%CI)** | **pval** | **Q_pval** | **egger_intercept** | **pleiotropy Pval** |
| **Stomatin gene** | 1.02(0.6,1.72) | 0.04 | 0.96 | 0.07 | 0.14 |

| **Table S2.** Results of Bayesian regression analysis of disease-free survival. | | | | | | | |
| --- | --- | --- | --- | --- | --- | --- | --- |
| **term** | **Estimate** | **Est.Error** | **lower** | **upper** | **Rhat** | **Bulk_ESS** | **Tail_ESS** |
| **LDH** | 0 | 0 | 0 | 0 | 1 | 4660 | 3700 |
| **Stomatin expression** | -2.27 | 0.15 | -2.55 | -1.99 | 1 | 1508 | 1895 |
| **Ann Arbor stage** | -0.21 | 0.03 | -0.27 | -0.14 | 1 | 1823 | 1681 |
| *Abbreviation: LDH, lactate dehydrogenase. | | | | | | | |

| **Table S3.** Results of Bayesian regression analysis of overall survival. | | | | | | | |
| --- | --- | --- | --- | --- | --- | --- | --- |
| **term** | **Estimate** | **Est.Error** | **lower** | **upper** | **Rhat** | **Bulk_ESS** | **Tail_ESS** |
| **LDH** | 0 | 0 | 0 | 0 | 1 | 4510 | 2942 |
| **Stomatin expression** | -1.43 | 0.12 | -1.67 | -1.18 | 1 | 1365 | 1624 |
| **Ann Arbor stage** | -0.22 | 0.03 | -0.28 | -0.16 | 1 | 1697 | 1435 |
| *Abbreviation: LDH, lactate dehydrogenase. | | | | | | | |
